# Inhibition of astroglial hemichannels ameliorates infrasonic noise induced short-term learning and memory impairment

**DOI:** 10.1186/s12993-023-00226-7

**Published:** 2023-12-18

**Authors:** Wei Zhang, Jue Yin, Bei-Yao Gao, Xi Lu, Ya-Jing Duan, Xu-Yan Liu, Ming-Zhen Li, Shan Jiang

**Affiliations:** 1 Teaching and Evaluation Center of Air Force Medical University, Xi’an, 710032 China; 2 Department of Rehabilitation Medicine, The China-Japan Friendship Hospital, No.2 Ying Hua Yuan East Street, Beijing, 100029 People’s Republic of China

**Keywords:** Infrasound, Learning and memory, Glutamate, ATP, Cx43 hemichannels

## Abstract

As a kind of environmental noise, infrasonic noise has negative effects on various human organs. To date, research has shown that infrasound impairs cognitive function, especially the ability for learning and memory. Previously, we demonstrated that impaired learning and memory induced by infrasound was closely related with glia activation; however, the underlying mechanisms remain unclear. Connexin 43 hemichannels (Cx43 HCs), which are mainly expressed in hippocampal astrocytes, are activated under pathological conditions, lending support to the hypothesis that Cx43 HCs might function in the impaired learning and memory induced by infrasound. This study revealed that that blocking hippocampal Cx43 HCs or downregulating hippocampal Cx43 expression significantly alleviated impaired learning and memory induced by infrasound. We also observed that infrasound exposure led to the abundant release of glutamate and ATP through Cx43 HCs. In addition, the abundant release of glutamate and ATP depended on proinflammatory cytokines. Our finds suggested that the enhanced release of ATP and glutamate by astroglial Cx43 HCs may be involved in the learning and memory deficits caused by infrasound exposure.

**Supplementary Information:**

The online version contains supplementary material available at 10.1186/s12993-023-00226-7.

## Introduction

Infrasound is a kind of environmental noise with a frequency below 20 Hz that is hardly heard by the human ear [1]. Infrasound can be produced by many natural sources, such as ocean waves, wind, and volcanic eruptions [2]. Infrasound is mainly responsible for vibroacoustic disease (VAD) [3]. Currently, with the widespread use of electronic appliances, increasing amounts of infrasound are produced in our environment [4]. Infrasound is characterized by reduced attenuation during propagation over long distances, strong vibration/penetration and protection difficulties, which make infrasound a significant type of environmental pollutant and a new threat to public health [5, 6].

Infrasound has adverse effects on multiple organs of humans and rodents, and the central nervous system (CNS) is particularly vulnerable to infrasound exposure (IE) [7–9]. Infrasound can result in a series of symptoms in which learning and memory impairment is the most common [10].

As the center of learning and memory, the hippocampus showed excessive activation of astrocytes after IE [11–13]. This result suggested that astrocytes probably participate in the impairment of learning and memory. However, the underlying molecular mechanisms remain largely unclear. A characteristic feature of astrocytes is their high level of hemichannels (HCs), which are a marker of the astroglial functional state [14, 15] and Connexin43 (Cx43) is the primary molecular constituent of HCs in hippocampal astrocytes [16–18]. Accumulating evidence has confirmed the Cx43 HCs are involved in cognitive impairment under pathological conditions, such as Parkinson’s disease and neurodegenerative disorders [19–22]. Accordingly, the aim of the present study was to clarify the role of astrocytes and astroglial Cx43 HCs in the hippocampus during learning and memory impairment under IE. Based on pharmacological approaches, we found that blocking Cx43 HCs or downregulating Cx43 expression was effective to ameliorate learning and memory impairment in rats under IE.

As plasma membrane channels, Cx43 HCs allow the passage of gliotransmitters and provide a rapid communication between the internal and external environment [23, 24]. In vitro, we showed that IE promoted the excessive release of glutamate and ATP by Cx43 HCs. This response depended on proinflammatory cytokines. Thus, these results reveal a novel mechanism in the pathogenesis of learning and memory impairment by infrasound.

## Materials and methods

### Animals and grouping

Sprague–Dawley (SD) rats (male, 7–8 weeks old, weight: 220–250 g) were purchased from the Animal Center of the China-Japan Friendship Hospital. The experiment complied with the Guidelines for the Care and Use of Experimental Animals approved by the China-Japan Friendship Hospital. The rats (n = 4 per cage) were housed in polycarbonate cages with a 12-h light/dark cycle in a temperature-controlled room (24 ± 1 °C).

**Fig. 1:**
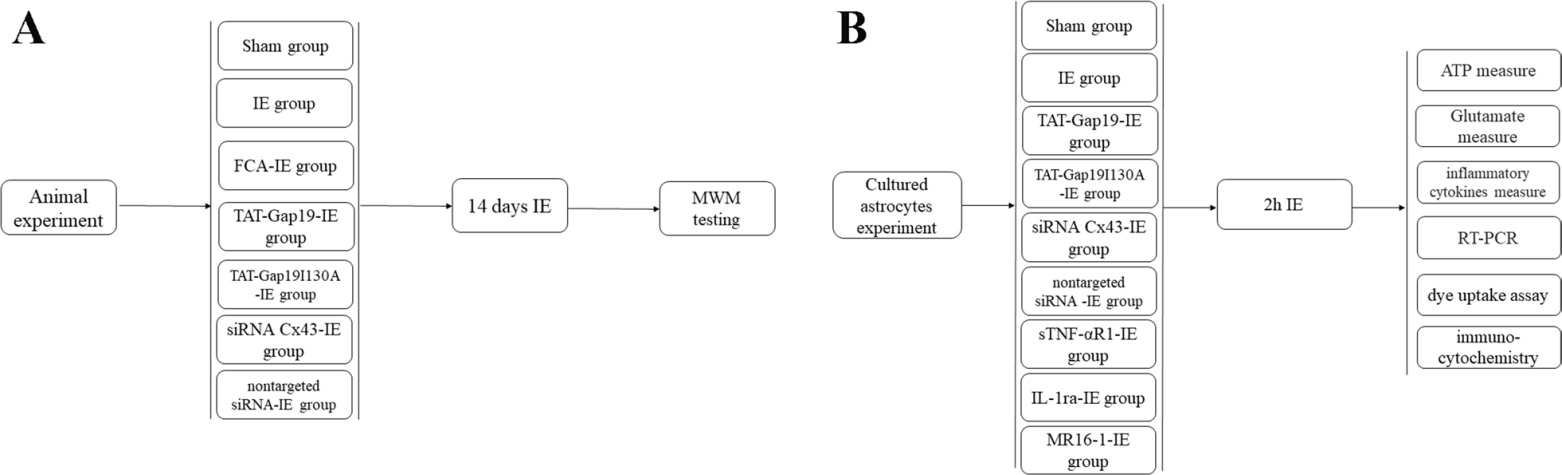
Flow chart of the experimental process. **A:** animal experimental process, **B:** experimental process in vitro

42 Rats were randomly assigned to the following groups, 6 rats in each group: Sham group, IE group, FCA (fluorocitrate)-IE group (FCA is an inhibitor of astrocytic activation), TAT-Gap19-IE group (TAT-Gap19 is a specific blocker of Cx43 HCs), TAT-Gap19I130A-IE group (TAT-Gap19I130A is an I130A-modified Gap19 analog), siRNA Cx43-IE group (siRNA Cx43 is a small interfering RNA (siRNA) targeting *Cx43*), and a nontargeted siRNA-IE group (nontargeted siRNA is a control siRNA that has no effect on *Cx43* expression). Figure 1A shows the animal experimental process.

### Infrasound exposure

As described in our previous study [25, 26], the infrasound system comprised an infrasound generator, a power amplifier, an infrasonic cabin, an infrasonic sensor, and a data collection system. The infrasound generator generates infrasonic waves at a sound pressure of 90–140 dB, with a frequency range of 2–20 Hz. During infrasound exposure, we monitored and analyzed the frequency and sound pressure level using the infrasonic sensor and data collection system.

According to previous studies, 16 Hz, 130 dB infrasound exposure obviously affected the learning and memory ability of rats [12, 25]. Therefore, a frequency of 16 Hz frequency and 130 dB sound pressure were used in this study. Rats were exposed to 16 Hz and 130 dB infrasound for 2 h every day for 14 days. The Sham group comprised rats that were placed in the infrasonic cabin for 2 h every day for 14 days but with no exposure to infrasound.

### Drugs used and their administration

**Fig. 2:**
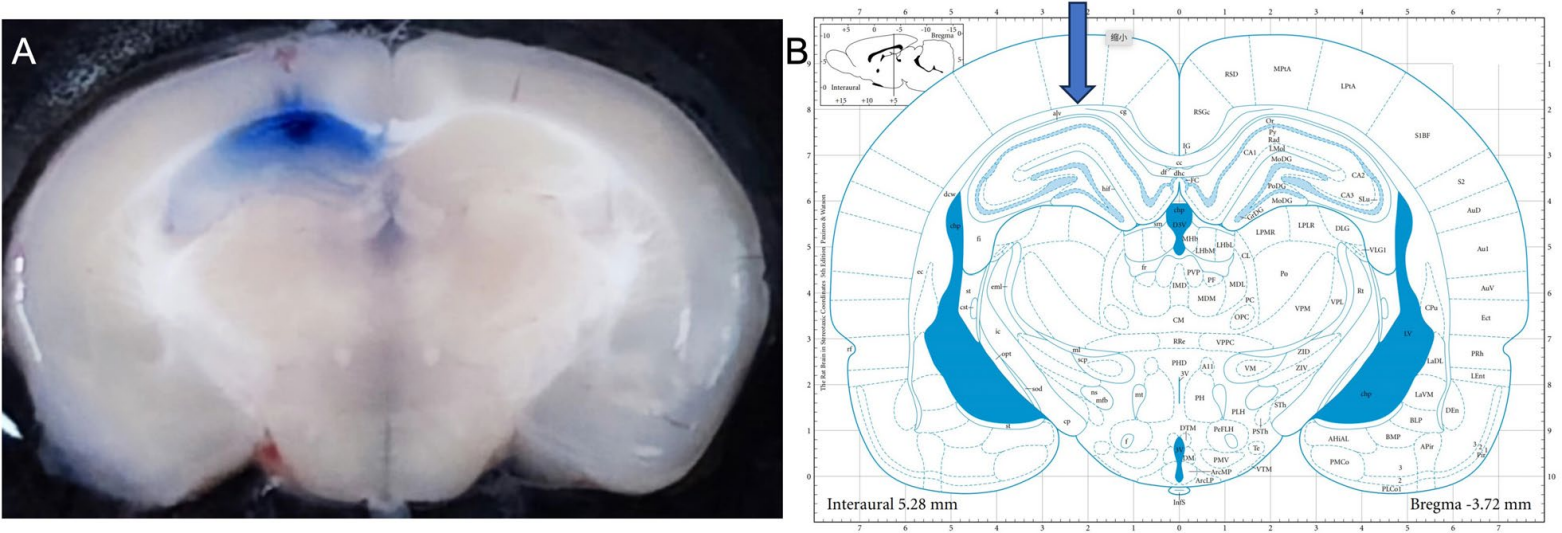
**A** Ink staining showed the microinjection site was hippocampus. **B** Schematic representation of microinjection into the hippocampus

We inserted two sterile polyethylene tubes into the rat bilateral hippocampus. Using a brain stereotaxic apparatus, we determined the coordinates of the injection points as follows: anteroposterior (AP): 2.8 mm from Bergma; mediolateral (ML): 1.5 mm from Bregma; dorsoventral (DV): 2.7 mm from the skull surface. Ink staining showed the microinjection site was hippocampus (Fig. 2).

To assess the function of astrocytes in IE-induced learning and memory impairment, FCA (Sigma-Aldrich, Cat#F9634) was used to inhibit astrocytic activation. The FCA solution was prepared as previously described and administered at 0.5 μL/side/rat, 1 nmol in 1 μL modified Ringer’s solution [27, 28].

We also used a short interfering RNA (siRNA, sequence: rGrCrArGrUrGrCrArCrArUrGrUrArArCrUrArArUrUrUrATT) to knockdown of *Cx43* expression to clarify the role of Cx43 HCs in IE-induced learning and memory impairment. The siRNA targeting *Cx43* was prepared according to previous study [29]. Briefly, a SMART-pool^*®*^ comprising four siRNA-duplexes targeting *Cx43* (400 ng, siCx43, Dharmacon Research) or nontargeted siRNA (control siRNA, Dharmacon Research) were mixed with INTERFERin^®^ (Polyplus-transfection), diluted in a saline solution (0.9%) containing 5% glucose at a final volume of 5μL, and left on ice for 20 min prior to injection. In normal rats, siRNA targeting Cx43 down-regulated about 30% Cx43 mRNA level in hippocampus (see supplementary material Fig. 1).

According to the experimental design, we used TAT-Gap19 (Sigma-Aldrich, Cat# SML2319, 0.5 μL/side/rat, 1 nmol in 1 μL of modified Ringer’s solution) to block Cx43 HCs. At the same time, TAT-Gap19I130A (0.5 μL/side/rat, 1 nmol in 1 μL of modified Ringer’s solution), an I130A-modified Gap19 analog, was used as a negative control peptide. Amino acid I130 forms hydrogen bonds that are important for Gap19 activity [30]. Therefore, TAT-Gap19I130A would have has no inhibitory effect Cx43 activity in HCs.

Two hours before IE, TAT-Gap19, TAT-Gap19I130A, or FCA were infused slowly through the polyethylene tubes, connected by PE-20 tubing to microsyringes driven by a microinfusion pump (CMA 400 Syringe Pump, CMA Microdialysis). Infusions were at a flow rate of 0.5 mL/min daily during IE, as described previously [31]. siRNA for *Cx43* or nontargeted siRNA (4 μL, respectively) were injected 30 min before IE, once every two days during IE at 0.5 μL/min.

### Learning and memory testing

At day 1 after 14 days infrasound exposure, spatial learning and reference/working memory was assessed using a Morris water maze (MWM) [32, 33]. The maze consisted of a dark circular tank with a diameter of 178 cm, which was filled with water to a depth of 37 cm and was separated into four quadrants (A–D). A plexiglass platform (diameter = 10.2 cm) was placed in quadrant C (the southeast quadrant) at approximately 28 cm from the wall of the pool and submerged at 2 cm below the waterline. Each animal was tested for 6 days. In this test, a 5-day testing block (acquisition trial, four trials each day) was performed to detect the rat's spatial learning ability. In each trial, the rats were put into a random quadrant, facing the wall. In 120 s of given time, we recorded the latency to the reach the platform. If the rats could not reach the platform, we physically guided them to it. After reaching it, the rats were kept on the platform for 30 s, followed by a 5-min rest before the next test. To determine the role of non-spatial factors, on the sixth day, the platform was raised to 2 cm above the waterline allowing the rats to see it. On the sixth day, a single probe trial was also carried out to assess memory retention. The platform was removed and the rats were allowed to explore the maze for 30 s. The detection indices were: the proportion of the duration of residence in the target quadrant (%) and the frequency of crossing the platform location.

### Astrocyte culture and grouping

The hippocampal astrocytes were cultured as in our previous study [34]. In brief, the rats (newborn SD rats (1 day)) were decapitated in an unanesthetized condition and their brains were removed. The hippocampus was carefully isolated, digested using 2.5% trypsin, and dissociated into a single-cell suspension in Dulbecco’s modified Eagle’s medium (DMEM; Invitrogen, Cat#11,965,092) containing 10% fetal bovine serum (FBS; Invitrogen, Cat#10099141C), streptomycin (0.2 mg/mL), and penicillin (80 units/mL). We plated the dissociated cells on plastic culture dishes with glass coverslips and incubated them at 37 ℃ in a humidified environment in the presence of 5% CO_2_. After five days, cellular debris was removed by washing with DMEM. Finally, the samples were shaken overnight at 37 ℃ to remove microglia and oligodendrocytes. Immunofluorescence cytochemical staining (using anti-glial fibrillary acidic protein (GFAP; Sigma-Aldrich, Cat#C9205, RRID:AB_ 476889) showed that culture purity exceeded 95% (see supplementary material Fig. 2). After 12–15 days of culture, these astrocytes were prepared for subsequent experiments.

In vitro, the cultured astrocytes were randomly assigned to following groups: Sham group, IE group, TAT-Gap19-IE group, TAT-Gap19I130A-IE group, siRNA Cx43-IE group, nontargeted siRNA-IE group, sTNF-αR1-IE group (sTNF-αR1 is a soluble form of the tumor necrosis factor alpha receptor (TNF-α receptor)), IL-1ra-IE group (IL-1ra is a recombinant antagonist for the interleukin (IL)-1β receptor)), and the MR16-1-IE group (MR16-1 is an anti-IL-6 receptor (IL-6R) antibody). For every group, the purified astrocytes were plated at a density of 5 × 10^4^ cells/cm^2^ onto 6 glass coverslips (for immunocytochemistry) or 1 × 10^6^cells/cm^2^ in 35-mm dishes (for ATP/glutamate/inflammatory cytokines measure, quantitative real-time reverse transcription-PCR, dye uptake assay. 6 dishes for every experiment). Thus, the sample size was 6 for every experiment. Figure 1B shows the experimental process in vitro.

### Astrocyte treatment with infrasound and chemical reagents

To produce the IE model in cultured astrocytes, the glass coverslips or dishes (cultured astrocytes) were placed into infrasonic chamber and exposed to 16 Hz and 130 dB infrasound for 2 h [8, 12, 26].

For 1 h before IE, the astrocytes were treated with the following pharmacological agents, separately: TAT-Gap19 (100 μM), TAT-Gap19I130A (100 μM), sTNF-αR1 (R&D Systems, Cat#425-R1-050; 200 ng/mL), IL-1ra (Sigma-Aldrich, Cat#SRP6006; 200 ng/mL), MR16-1 (Chugai Pharmaceutical Co.Ltd, 200 ng/mL). siRNA Cx43 or nontargeted siRNA was transfected 24 h before IE treatment.

### Dye uptake assay

A dye uptake assay was performed in cultured astrocytes to measure HC activity was as previously described [35]. After IE, cultured astrocytes were incubated with the HC permeable fluorescent tracer ethidium bromide (Etd; Sigma-Aldrich, Cat#46067) for 10 min at 37 ℃ and at 4 μM final concentration. Following the 10 min incubation with Etd, astrocytes were washed five times with Hank's balanced salt solution (HBSS, Invitrogen, Cat# 24020117), fixed with 4% paraformaldehyde, and detected using a microscope (Olympus). The fluorescence intensity was calculated as follows: the fluorescence intensity of respective astrocytes (*F*) minus the fluorescence intensity of the background (*F0*).

### Measurement of glutamate in extracellular fluid

After IE, culture media of astrocytes was isolated by centrifugation at 250 × *g* for 4 min, followed by high pressure liquid chromatography (HPLC, Waters) was used to assess the level of glutamate in the extracellular fluid [34].

Briefly, extracellular fluid was derivatized using *o*-phthalialdehyde (OPA; Sigma-Aldrich, Cat#P0657). OPA-derivatives were passed through a C18 reverse-phase Adsorbosphere OPA-HR column (Alltech), and eluted using mobile phase A (a mixture of odium acetate, dioxane, and isopropanol, 92.5/4.5/3.0 (v/v)) and mobile phase B (a mixture of methanol, dioxane, and isopropanol, 97/1.5/1.5 (v/v)). A fluorescence detector was used to detect the glutamate level and the excitation and emission wavelengths were 338 and 450 nm, respectively.

### Measurement of ATP in extracellular fluid

A luciferin/luciferase bioluminescence assay kit (Sigma-Aldrich, Cat#119,107) was used to detect extracellular ATP levels. Standard curves were used to calculate the amounts of ATP in samples, which normalized according to the protein concentration, as determined using a Bio-Rad protein assay (Bio-Rad).

#### Measurement of inflammatory cytokines in extracellular fluid

The culture media was collected after IE and assayed simultaneously to detect the levels of IL-1β, IL-6, and TNF-α using enzyme-linked immunosorbent assays (ELISAs), according to the supplier's instructions (Nanjing Jiancheng Bioengineering Institute, IL-1β: Cat# H002-1-2, IL-6: Cat# H007-1-2, TNF-α: Cat# H052-1-2).

#### Immunocytochemistry

Astrocytes were cultured on coverslips, and then incubated with a mouse monoclonal antibody recognizing GFAP (Sigma-Aldrich, Cat#C9205, RRID:AB_ 476889) and rabbit polyclonal antibodies recognizing Cx43 (Sigma-Aldrich, Cat#SAB4501173, RRID:AB_ 476889) at 4 °C overnight. After washing, the astrocytes were incubated with secondary antibodies for 2 h at room temperature. Immunostaining signals were detected using laser confocal scanning microscopy (IX-70; Olympus).

#### Quantitative real-time reverse transcription PCR analysis

*Cx43* expression in the hippocampus or cultured astrocytes was assessed using quantitative real-time reverse transcription-PCR (qRT-PCR). The Trizol reagent (Invitrogen, Cat# 15596026) was used to extract total RNA from rat hippocampus or cultured astrocytes. The total RNA was converted to cDNA using reverse transcription. The ABI 7901HT sequence detection system (Applied Biosystems) with a Power SYBR green PCR Master Mix kit (Takara) were used to carry out the qPCR step with the cDNA as the template. The assays used the following primers: *Cx43*: forward primer: CGTGGAGATGCACCTGAA, reverse primer: CCACTGGATGAGCAGGAA, *Gapdh* (encoding glyceraldehyde-3-phosphate dehydrogenase): forward primer: AGCCCAGAACATCATCCCTG, reverse primer: CACCACCTTCTTGATTGTCATC. *Gapdh* expression was used to normalize all the data, which was further normalized to the expression in the control.

#### Statistical analysis

The quantitative results are presented as the mean ± the standard deviation (SD). Normality was tested using the Kolmogorov–Smirnov test. A t test was used to compare data between two groups. Comparisons among multiple groups used one-way analysis of variance (ANOVA), followed by Tukey’s post-hoc analysis. The statistical analyses were carried out using GraphPad Prism 9.0 software (GraphPad Inc). A p value < 0.05 was considered to indicate statistical significance.

## Results

### IE Impairs Hippocampal Learning and Memory in Rats

The MWM test was used to determine the effect of IE on spatial learning and reference/working memory.

**Fig. 3:**
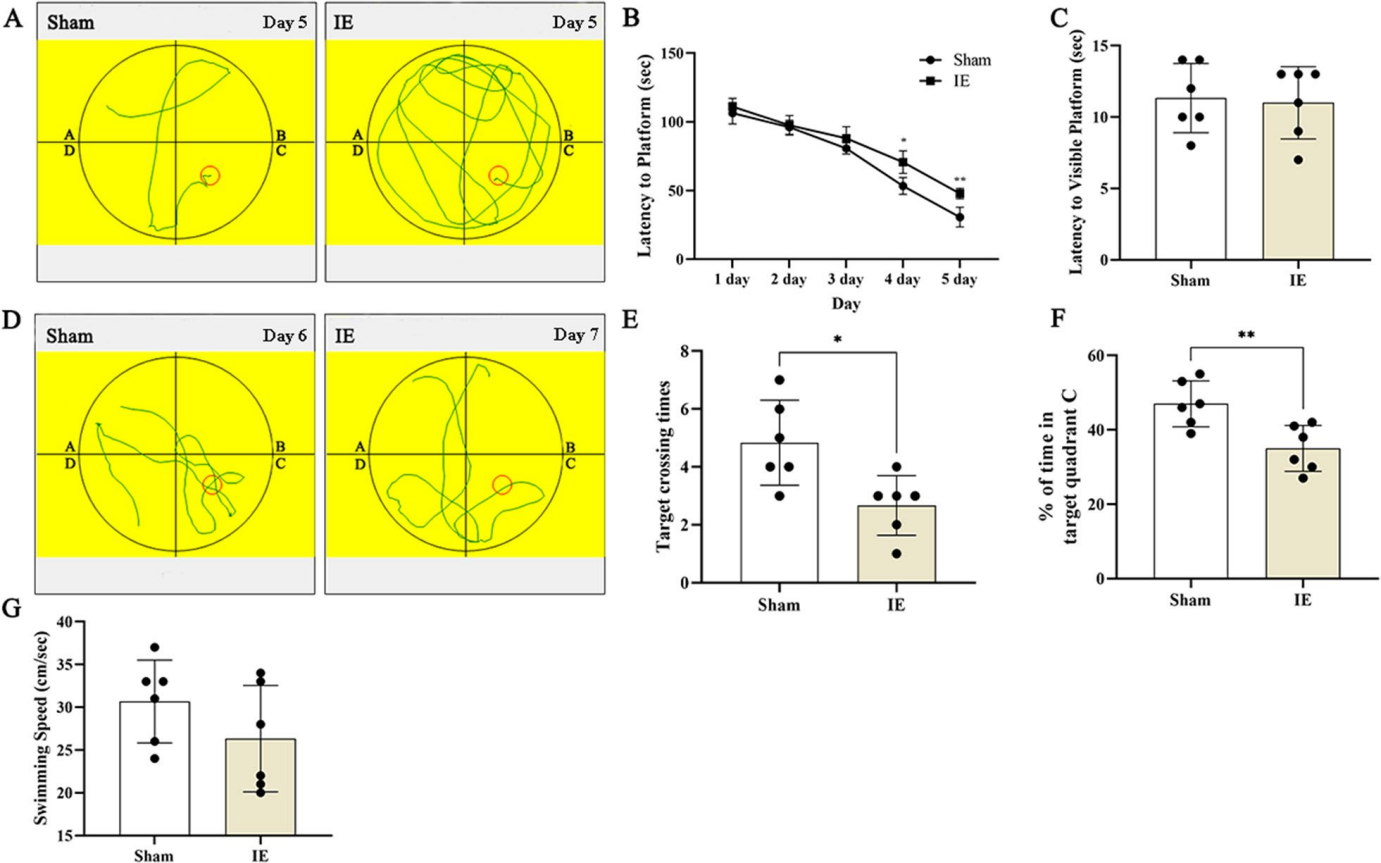
**A** An example of acquisition testing of MWM. The rats subjected to IE had more complex motion track to find the submerged platform than the rats in the Sham group. Red circle indicates the platform. **B** At day 4 or day 5, the rats in the Sham group exhibited shorter latency to platform when compared with the rats in the IE group. **C** There was no difference in latency to visible platform between two groups. **D** An example of probe trial of MWM. The platform was removed and the motion track showed that the rats in the IE group had less time spent in target quadrant (quadrant C) and less times crossing the removed platform location than the rats in the Sham group. Red circle indicates the location of platform. **E** The rats in the IE group had less time spent in target quadrant (quadrant C) when compared with the rats in the Sham group. **F** The rats in the Sham group had more target crossing times than the rats in the IE group. **G** There was no difference in swimming speed between two groups. ^*^p < 0.05, ^**^p < 0.01. Each value represents means ± SD, n = 6

The rats subjected to IE took longer to reach the submerged platform with complex motion track than the rats in the Sham group on day 4 and 5, indicating that IE impaired spatial learning (Fig. 3A, B; day 4: t = 4.153, p = 0.0117, df = 10; day 5: t = 5.144, p = 0.0050, df = 10). On day 6, the Sham rats and rats subjected to IE showed no significant differences in latency to reach the visible platform (Fig. 3C; t = 0.2331, p = 0.8204, df = 10), which confirmed that the differing escape latencies were not related to differences in vision. These findings implied that IE impaired the rats' spatial learning ability.

On the final day of the MWM test, we removed the platform and recorded the time spent in quadrant C and the frequency of crossing the platform zone to analyze reference and/or working memory retention. Compared with the IE-treated rats, the rats in the Sham group showed better memory retention (higher percentage of allotted time spent in quadrant C (Fig. 3D, E; t = 3.363, p = 0.0072, df = 10). This suggested that IE impaired the rats' memory. Similar results were obtained in the analysis of the frequency of crossing the platform zone, i.e., the IE-treated rats crossed the platform zone less frequently than the Sham group rats (Fig. 3D, F; t = 2.951, p = 0.0145, df = 10). The two groups showed no differences in swimming speed (Fig. 3G; t = 1.347, p = 0.2078, df = 10), indicating that motor defects did not contribute to the observed differences.

### Inhibiting astrocytes or Cx43 hemichannels ameliorates IE-induced learning and memory impairment

**Fig. 4:**
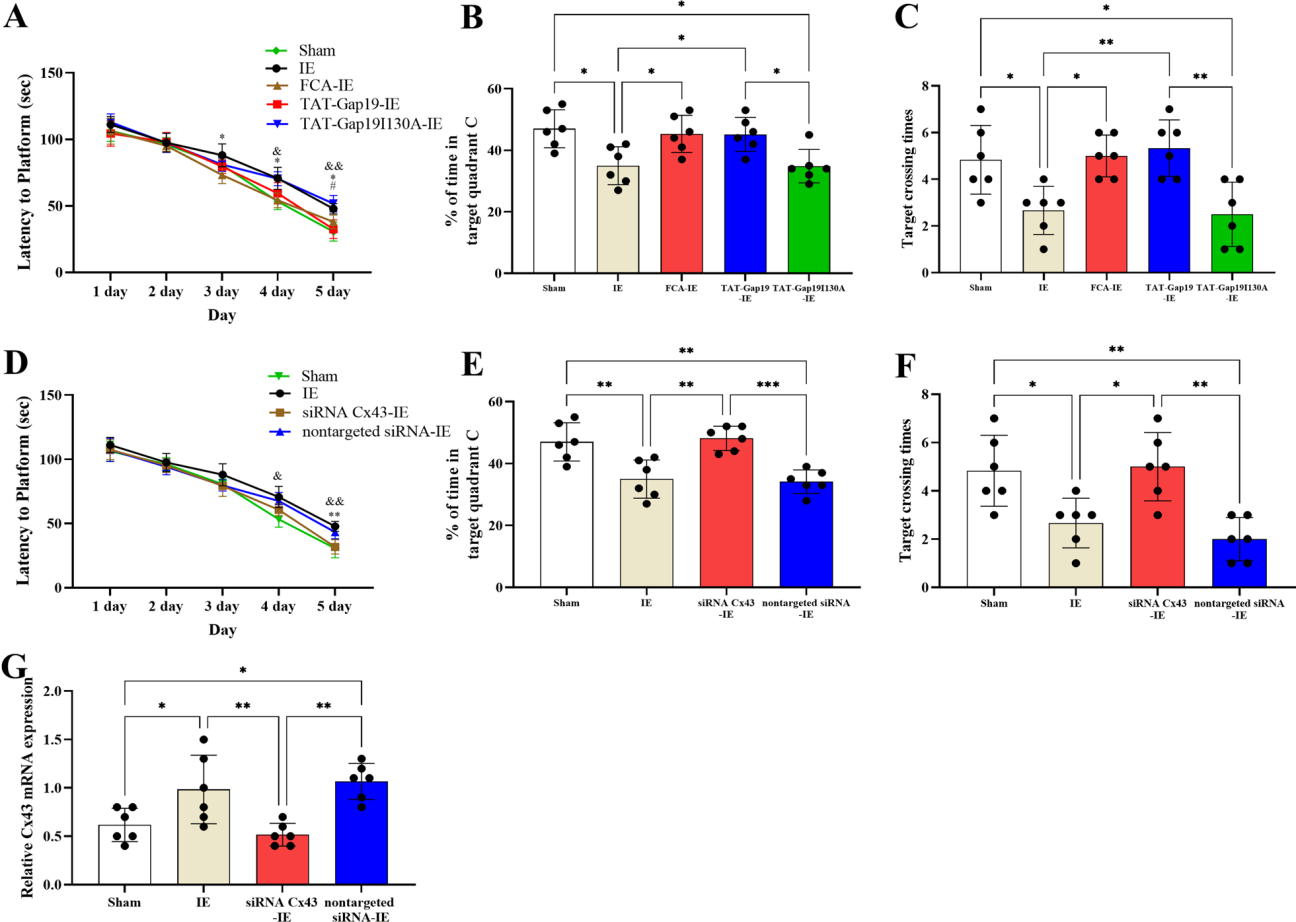
**A** At day 3, day 4 or day 5, the rats in the IE group had the longer latency to platform than the rats in the FCA-IE group. At day5, the rats in the IE group had the longer latency to platform than the rats in the TAT-Gap19-IE group. IE *vs.* FCA-IE group: ^*^p < 0.05; IE *vs.* TAT-Gap19-IE group: ^#^p < 0.05; IE *vs.* Sham group: ^&^p < 0.05, ^&&^p < 0.01. **B** The rats in the FCA-IE or TAT-Gap19-IE group spent more time in target quadrant C compared with the rats in the IE group. ^*^p < 0.05. **C** The rats in the FCA-IE or TAT-Gap19-IE group had more target crossing times when compared with the rats in the IE group. ^*^p < 0.05, ^**^p < 0.01. **D** At day 5, the rats in the IE group had the longer latency to platform than the rats in the siRNA Cx43-IE group. IE *vs.* siRNA Cx43-IE group: ^**^p < 0.01; IE *vs.* Sham group: ^&^p < 0.05, ^&&^p < 0.01. **E** The rats in the siRNA Cx43-IE group spent more time in target quadrant C compared with the rats in the IE group. ^**^p < 0.01, ^***^p < 0.001. **F** The rats in the siRNA Cx43-IE group had more target crossing times when compared with the rats in the IE group. ^*^p < 0.05, ^**^p < 0.01. **G** The rats in the siRNA Cx43-IE group had lower expression of Cx43 mRNA when compared with the rats in the IE or nontargeted siRNA-IE group. ^*^p < 0.05,^**^p < 0.01.Data represents means ± SD, n = 6

IE increases GFAP expression, which is a marker of astrocyte activation [12, 13]. Accordingly, we explored whether astrocytes were responsible for the impaired learning and memory induced by IE. Microinjection of FCA, an astrocyte inhibitor, into the hippocampus of the IE-treated rats decreased their escape latencies, increased the time spent in target quadrant C, and increased the frequency of crossing the platform zone to the sham group level (Fig. 4A, B, C; Latency to platform: day 3: F(4, 25) = 4.608, p = 0.0063, Post-hoc analysis: IE *vs.* FCA-IE group: p = 0.0449; day 4: F(4, 25) = 9.634, p < 0.0001, Post-hoc analysis: IE *vs.* FCA-IE group: p = 0.0185; day 5: F(4, 25) = 14.32, p < 0.0001, Post-hoc analysis: IE *vs.* FCA-IE group: p = 0.0265; % of time in target quadrant C: F(4, 25) = 6.289, p = 0.0012, Post-hoc analysis: IE *vs.* FCA-IE group: p = 0.0397; Target crossing times: F(4, 25) = 7.579, p = 0.0004, Post-hoc analysis: IE *vs.* FCA-IE group: p = 0.0361). These findings suggested that IE induced impaired learning and memory substantially via astrocytes.

The primary molecular marked and constituent of HCs in hippocampal astrocytes is Cx43 [36]. Cx43 HCs have a role in cognitive impairment under pathological conditions [15, 37, 38]. Accordingly, we investigated the role of Cx43 HCs in IE-induced learning and memory impairment. We used Gap19, a blocking peptide of Cx43 intracellular loop domains, to block Cx43 HCs. To increase Gap19's cell membrane permeability, we coupled it to the TAT membrane translocation motif (TAT-Gap19), whose C-terminal end is intracellularly located [39]. Microinjection of TAT-Gap19 into hippocampus effectively decreased the IE-induced learning and memory impairment (Fig. 4A, B, C; Latency to platform: day 5: Post-hoc analysis: IE *vs.* TAT-Gap19-IE group: p = 0.0108; % of time in target quadrant C: Post-hoc analysis: IE *vs.* TAT-Gap19-IE group: p = 0.0442; Target crossing times: Post-hoc analysis: IE *vs.* TAT-Gap19-IE group: p = 0.0068), suggesting that Cx43 HCs might mediate the learning and memory impairment caused by IE. In addition, TAT-Gap19I130A, which is structurally similar to TAT-Gap19 but has no effect on HCs, did not alter IE's effect on learning and memory (Fig. 4A, B, C).

After microinjection of the siRNA targeting *Cx43*, we observed an approximately 50% decline in the level of *Cx43* mRNA in the hippocampus. (Fig. 4G; F (3, 20) = 8.584, p = 0.0006, Post-hoc analysis: IE *vs.* siRNA-Cx43-IE group: p = 0.0007). This result also revealed IE increased the Cx43 expression in the hippocampus (Fig. 4G; Post-hoc analysis: Sham *vs.* IE group: p = 0.0484). As expected, impaired learning and memory by IE was markedly decreased by siRNA-Cx43 treatment (Fig. 4D, E, F; Latency to platform: day 5: F(3, 20) = 13.90, p < 0.0001, Post-hoc analysis: IE *vs.* siRNA-Cx43-IE group: p = 0.0013; % of time in target quadrant C: F(3, 20) = 12.79, p = 0.0002, Post-hoc analysis: IE *vs.* siRNA-Cx43-IE group: p = 0.0014; Target crossing times: F(3, 20) = 9.162, p = 0.0005, Post-hoc analysis: IE *vs.* siRNA-Cx43-IE group: p = 0.0176), whereas treatment with the nontargeted siRNA had no effect on IE-induced learning and memory impairment.

Taken together, our results indicated that astroglial Cx43 HCs mediated IE-induced learning and memory impairment.

### IE Causes Cx43 hemichannels-mediated glutamate and ATP release from astrocytes

**Fig. 5:**
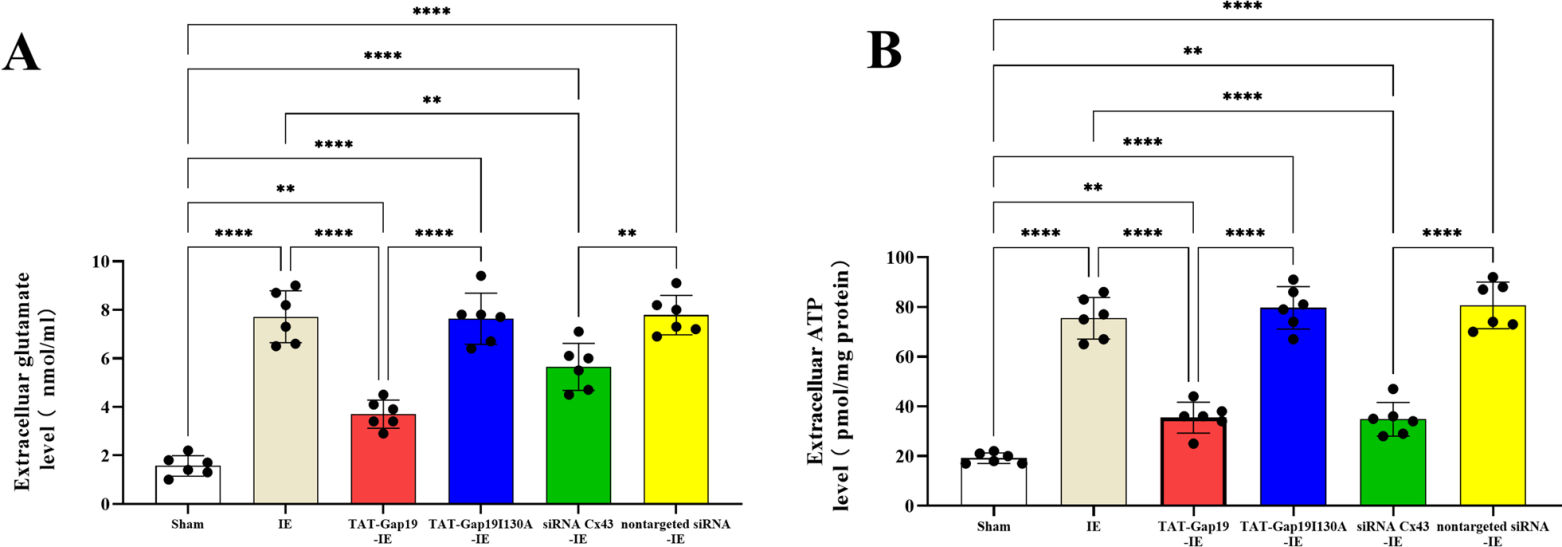
**A** The extracelluar glutamate level in the IE group was higher than that in the sham, TAT-Gap19-IE or siRNA Cx43-IE group. The extracelluar glutamate level in the in the TAT-Gap19-IE group was lower than that in the TAT-Gap19I130A-IE group. The extracelluar glutamate level in the siRNA Cx43-IE group was lower than that in the nontargeted siRNA-IE group. **B** The extracelluar ATP level in the IE group was higher than that in the sham, TAT-Gap19-IE or siRNA Cx43-IE group. The extracelluar ATP level in the TAT-Gap19-IE group was lower than that in the TAT-Gap19I130A-IE group. The extracelluar ATP level in the in the siRNA Cx43-IE group was lower than that in the nontargeted siRNA-IE group. ^**^p < 0.01, ^****^p < 0.0001. Data represents means ± SD, n = 6

Our results showed that astroglial Cx43 HCs are involved in the learning and memory impairment induced by IE; therefore, the detailed mechanism in primary hippocampal astrocytes was investigated. Past studies demonstrated that Cx43 HCs exert their effects by releasing neuroactive substances, such as ATP and glutamate, now termed “gliotransmitters” [40]. We examined the glutamate level in extracellular fluid after IE using HPLC. IE resulted in a significant increase in the extracellular glutamate level (to 7.72 nmol/mL) compared with that in the control astrocytes (Sham group: 1.57 nmol/mL) representing an approximately fivefold increase (F(5, 30) = 54.73, p < 0.0001, Post-hoc analysis: Sham vs. IE group: p < 0.0001; Fig. 5A). Similarly, the extracellular ATP level was increased by fourfold compared with that in the control group (from 19.17 pmol/mg protein to 75.5 pmol/mg protein; (F(5, 30) = 84.52, p < 0.0001, Post-hoc analysis: Sham *vs*. IE group: p < 0.0001; Fig. 5B). These findings suggested that IE induced the excessive release of glutamate and ATP.

Additionally, TAT-Gap19 partially abrogated the release of glutamate caused by IE, as indicated by the differences between the IE group and the TAT-Gap19-IE, Sham, and TAT-Gap19-IE group. The glutamate level decreased to about 3.70 nmol/mL (Post-hoc analysis: TAT-gap19-IE vs. IE group: p < 0.0001; TAT-gap19-IE vs. Sham group: p = 0.0019; Fig. 5A). Similarly, siRNA-mediated knockdown of *Cx43*, but not the nontargeted siRNA, also partly inhibited the release of glutamate induced by IE. The glutamate level decreased to about 5.65 nmol/mL (Post-hoc analysis: siRNA Cx43-IE vs. IE group: p = 0.0028; siRNA Cx43-IE vs. Sham group: p < 0.0001; Fig. 5A). TAT-Gap19 also strongly reduced the IE-induced release of ATP. The ATP level in the TAT-gap19-IE group was about 35.50 pmol/mg protein (Post-hoc analysis: TAT-gap19-IE vs. IE group: p < 0.0001; TAT-gap19-IE vs. Sham group: p = 0.0065; Fig. 5B). *Cx43* knockdown by siRNA-Cx43, but not the nontargeted siRNA, also markedly abrogated IE-induced ATP release. The ATP level in the siRNA Cx43-IE group was about 34.83 pmol/mg protein (Post-hoc analysis: siRNA Cx43-IE vs. IE group: p < 0.0001; siRNA Cx43-IE vs. Sham group: p = 0.0098; Fig. 5B). These results suggested that Cx43 HCs contribute to the excessive release of glutamate and ATP. As excitatory neurotransmitters, excessive signaling by glutamate and ATP is primarily responsible for the impairment of hippocampal neurons, resulting in impaired learning and memory ability [41].

### IE-induced release of glutamate and ATP depends on proinflammatory cytokines

**Fig. 6:**
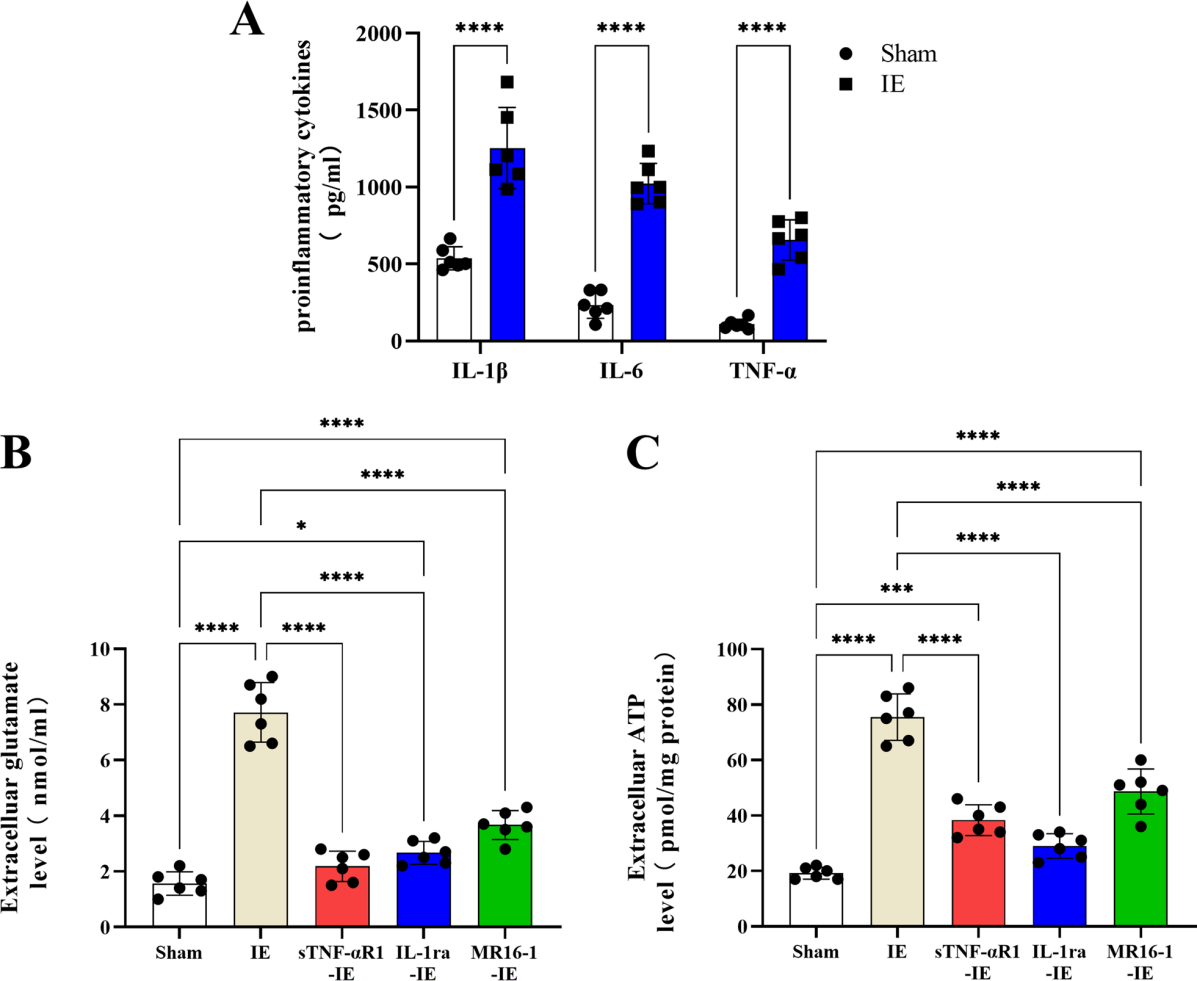
**A** The proinflammatory cytokines level in the IE group was higher than that in the sham group. **B** The extracelluar glutamate level in the IE group was higher than that in the sham, sTNF-αR1-IE, IL-1ra-IE or MR16-1-IE group. **C:** The extracelluar ATP level in the IE group was higher than that in the sham, sTNF-αR1-IE, IL-1ra-IE or MR16-1-IE group. ^*^p < 0.05, ^***^p < 0.001, ^****^p < 0.0001. Data represents means ± SD, n = 6

Under pathological condition, astrocytes release copious amounts of inflammatory cytokines, resulting in modulation of astrocyte's molecular, morphological, and functional properties via autocrine/paracrine signaling [42]. Consequently, ELISAs were used to examine the changes of proinflammatory cytokines (IL-1β, IL-6, and TNF-α) in the extracellular fluid after exposure of astrocytes to IE. The In the extracellular fluid of IE-treated astrocytes, the levels of IL-1β, IL-6, and TNF-α were increased by twofold, fourfold, and sixfold, respectively, compared with those in the Sham group (IL-1β: t = 6.422, df = 10, p < 0.0001; IL-6: t = 12.31, df = 10, p < 0.0001; TNF-α: t = 9.982, df = 10, p < 0.0001; Fig. 6A). These results indicated that IE induces the release of proinflammatory cytokines from astrocytes.

We also tested whether the increased release of proinflammatory cytokines was involved in the glutamate and ATP release by astrocytes induced by IE. Astrocytes were pre-treated using a soluble form of the TNF-α receptor that binds TNF-α (sTNF-αR1), a recombinant antagonist for the IL-1β receptor (IL-1ra) or anti-IL-6 receptor (IL-6R) antibodies (MR16-1). All three pre-treatments strongly reduced the release of glutamate and ATP evoked by IE in comparison with the controls (ATP: F(4, 25) = 73.60, p < 0.0001, Post-hoc analysis: all p < 0.0001; glutamate: F(4, 25) = 86.91, p < 0.0001, Post-hoc analysis: all p < 0.0001Fig. 6B, C). Thus, the release of proinflammatory cytokines seems to be vital for the excessive release of glutamate and ATP induced by IE.

### IE Activates astrocytes in culture

**Fig. 7:**
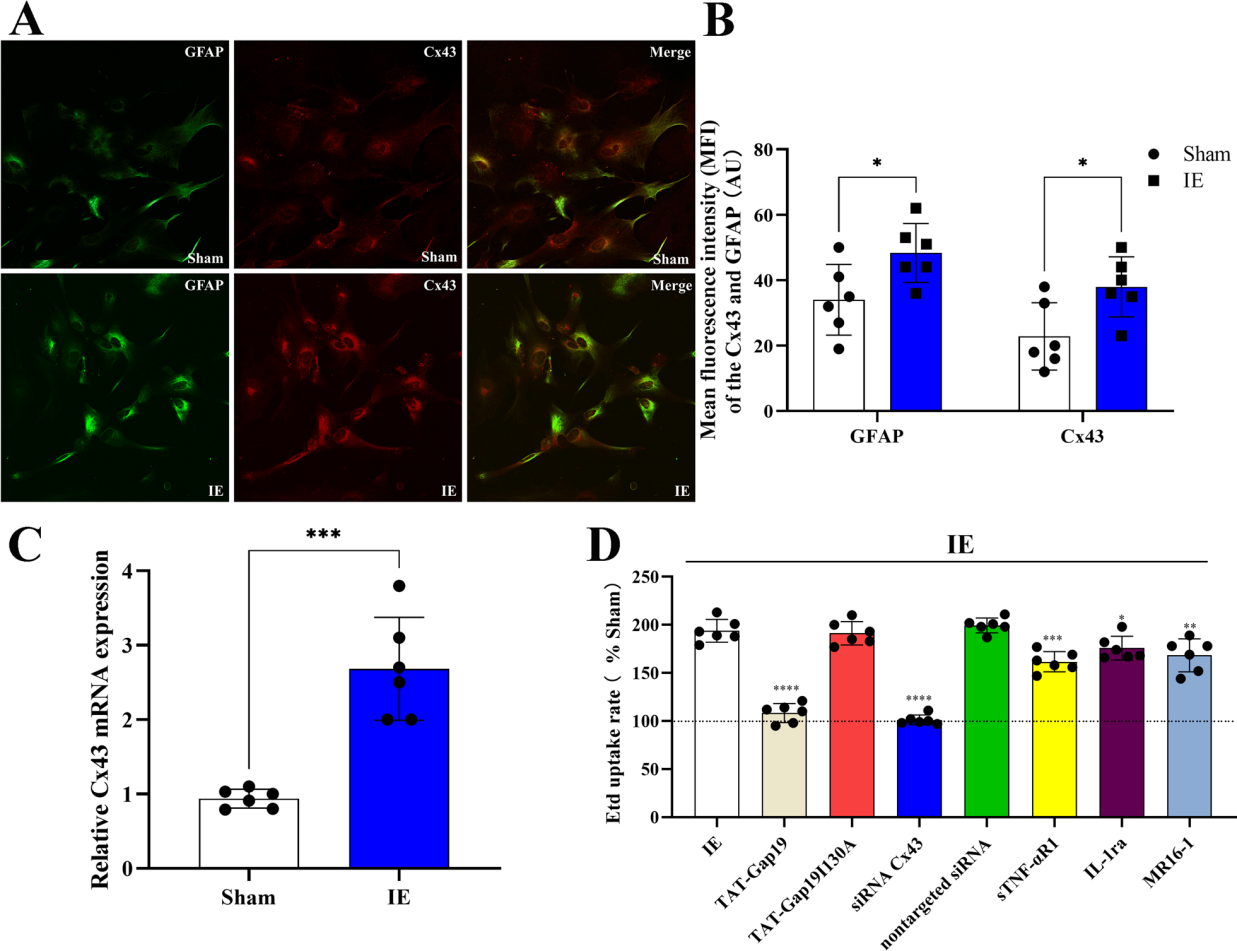
**A**, **B** The fluorescence intensity of the Cx43 or GFAP in the Sham group was lower than that in the IE group. **C** The Cx43 mRNA expression in the Sham group was lower than that in the IE group. **D** The Etd uptake ratio was lower in the TAT-Gap19-IE, siRNA Cx43-IE, sTNF-αR1-IE, IL-1ra-IE or MR16-1-IE group when compared with that in the IE group. ^*^p < 0.05, ^**^p < 0.01, ^***^p < 0.001,^****^p < 0.0001. Data represents means ± SD, n = 6

Our findings indicated that the release of ATP and glutamate via Cx43 HCs on cultured astrocytes is induced by IE. Further immunocytochemistry experiments showed a broad distribution of Cx43 over cultured hippocampal astrocytes (Fig. 7A). The mean fluorescence intensity (MFI) of the Cx43 signal was 22.83 ± 10.28 (AU) in the control group, which was used as the baseline value. Compared with that in the Sham group, the MFI in the IE group increased significantly to 38.00 ± 9.19 (AU) (Sham *vs*. IE group: t = 2.667, df = 10, p = 0.0296, Fig. 7A, B). In addition, IE increased the expression of the astrocyte marker GFAP (Sham *vs*. IE group: t = 2.521, df = 10, p = 0.0406, Fig. 7A, B). These findings suggested that IE activated astrocytes. Similar results were observed using qRT-PCR: compared with that in the Sham group, *Cx43* mRNA expression increased in the IE group (Sham *vs*. IE group: t = 6.083, df = 10, p = 0.0001, Fig. 7C).

The rate of Etd uptake was used to evaluate the functional state of Cx43 HCs. Etd can only cross the astrocyte plasma membrane via specific HCs. After IE, we detected a rapid increase (by twofold) in the Etd uptake rate by astrocytes compared with that of the Sham group astrocytes (Fig. 7D). This result suggested that IE triggered the opening of HCs. TAT-Gap19 or siRNA-Cx43, but not TAT-Gap19I130A or the nontargeted siRNA, effectively abolished the increased Etd uptake caused by IE (F (7, 40) = 67.08, p < 0.0001, Post-hoc analysis:TAT-Gap19-IE *vs*. IE group: p < 0.0001; TAT-Gap19I130A-IE *vs*. IE group: p = 0.9994; siRNA Cx43-IE vs IE group: p < 0.0001; nontargeted siRNA-IE *vs*. IE group: p = 0.9204; Fig. 7D), indicating that IE opened Cx43 HCs. Interestingly, when we applied sTNF-αR1, IL-1ra or MR16-1, the opening of Cx43 HCs was inhibited (sTNF-αR1-IE *vs*. IE group: p = 0.0001; IL-1ra-IE *vs*. IE group: p = 0.0483; MR16-1-IE *vs*. IE group: p = 0.0023). These findings suggested that proinflammatory cytokines mediate the opening of Cx43 HCs, resulting in the excessive release of glutamate and ATP.

## Discussion

The findings reported herein demonstrate that infrasound induces impaired learning and memory, as suggested by the MWM test results. This is likely achieved by astroglial Cx43 HCs, since blocking Cx43 HCs alleviated this impairment. Moreover, in vitro, IE activates the opening of Cx43 HCs in primary cultured hippocampal astrocytes. This enhanced HC opening is mediated by proinflammatory cytokines, which then promote the excessive release of glutamate and ATP, which might the mechanism responsible for IE-induced impairment of learning and memory.

Herein, the MWM was used to evaluate the rats' ability for learning and memory. The MWM is used widely to evaluate spatial learning and memory dependent on the hippocampus and is intimately associated with long-term potentiation (LTP) [43, 44]. Damaged learning and memory increased escape latency, decreased the time in the target quadrant (C), and decreased the number of times the rat crossed the target crossing after IE, which were consistent with the results of previous studies [10, 12, 45]. Notably, the rat swimming speed was approximately the same speed in the pool among the groups, suggesting that their performance in the MWM was not disturbed by a locomotor factor. Moreover, impairment of land-based locomotor function is unrelated to swimming speed, which might also account for the independence from locomotor effects of learning and memory performance in the MWM.

Hippocampal microinjection of an astrocyte inhibitor, a Cx43 HCs blocker or an siRNA targeting *CX43* indicated that IE-induced impairment of learning and memory acts via astrocytic Cx43 HCs activity. This finding is congruent with some previous studies showing that dysregulation of HCs is linked with the progression of different neurodegenerative diseases characterized by destruction of learning and memory [46, 47]. However, we only verified the inhibitory effect of siRNA on Cx43 mRNA in the whole hippocampus not in selected regions. Thus, it's not clear which part works (CA1, CA2 or CA3?). We will carry out research on this point in the future. In present study, FCA was used to inhibit the activity of astrocyte. FCA is a metabolic poison that inhibits the tricarboxylic acid (TCA) enzyme aconitase and is selectively taken up by astrocytes in the brain [48, 49]. FCA can cause a reversible and selective disruption of astrocyte function [48, 50]. Consequently, FCA has been widely used to study the role of astrocytes in brain [48]. Our previous study has shown that astrocytes exhibited decreased expression of GFAP and morphological alteration characterized by small soma with slender processes [51]. In addition, our preliminary experiment showed microinjection procedure had no effect on the outcomes of MWM, which ruled out the interference of microinjection itself on our research findings.

In this study, we demonstrated that infrasound increased the opening of HCs using Etd uptake experiments. That IE-induced HCs activity was caused by Cx43 was revealed by decreased Etd uptake upon siRNA-mediated downregulation of protein CX43 levels in astrocytes. Etd uptake was also decreased by treatment with TAT-Gap19, a Cx43 mimetic peptide that blocks Cx43 HCs. The synthetic nonapeptide Gap19 corresponds to AAs 128–136 in the second half of the Cx43 CL, which forms part of the L2 region [52] Gap19 comprises the cell-membrane translocation motif KKFK, which aids plasma membrane permeability [53]. Gap19 is more selective in inhibiting Cx43 hemichannels compared to Gap26 and Gap27 without influencing Cx43 gap junctions [39]. Moreover, Gap19 was coupled to TAT, a membrane translocation motif, to augment its cell membrane permeability further, because the C-terminal tail is located intracellularly [39]. Our results support the increased opening of HCs opening in astrocytes under pathological exposure including restraint stress [54], epileptic seizures [55], hypertonic stimulation [34], spinal cord injury [56, 57], and acute infection [17, 58].

Gliotransmitters, vital transmitters during neuron-astrocyte communication, are released when Cx43 HC open [59, 60]. Past studies have shown that uncontrolled opening of HCs could lead to the excessive release of excitotoxic gliotransmitters [61]. Herein, we noted that IE enhanced the excessive glutamate and ATP release by astroglial Cx43 HCs. High concentrations of glutamate and ATP at the synaptic cleft could decreased neuronal survival by activating purinergic receptor P2X 7/N-Methyl D-Aspartate (NMDA) receptors [54]. This was also observed in neurons under various pathological conditions [62–64], which might constitute the major mechanism for learning and memory impairment after IE. In addition, excessive amounts of ATP released by HCs activated purinergic receptor P2Y1, which was also related to cognitive decline [65, 66]. Studies have suggested that downregulation of P2Y1 receptors leads to a neuroprotective effect [67]. Pannexin1 (PANX1) is believed to have similar functions to Cx43 HCs and can release gliotransmitters under pathological conditions [68]. Therefore, it is possible that Panx1 contributed the IE-promoted excessive glutamate and ATP release in the present study.

How does IE evoke Cx43 HCs opening? It has been shown that astrocytes release large amounts of inflammatory cytokines, which regulate astroglial properties at the molecular, morphological, and functional level, via their autocrine/paracrine signaling [42]. Herein, we showed that exposure to infrasound precipitated Cx43 HC opening, dependent on astrocytes releasing proinflammatory cytokines. However, we did not investigate the detailed signaling pathways. Cx43 HC opening dependent on cytokine production and subsequent activation of p38 mitogen activated protein kinase (MAPK)/inducible nitric oxide synthase (iNOS)/cytochrome C oxidase subunit 2 (COX2)/inorganic calcium ([Ca^2+^]i)-dependent pathways and purinergic/glutamatergic signaling is enhanced by α-synuclein. In our next study, we will investigate if these signaling pathways are involved in the proinflammatory cytokine-induced Cx43 HC opening under IE condition.

In addition to releasing glutamate, astrocytes also produce large amounts of functional glutamate transporters to complete glutamate uptake [69]. Pathological conditions, e.g. ischemic injury, could decrease the expression of glutamate transporters in astrocytes [70], which resulted in the decreased of uptake of glutamate by astrocytes, thus glutamate levels in the extracellular fluid. These can probably explain why blockage of Cx43 HCs cannot entirely, but only partly, inhibit the IE-induced increase in the glutamate level.

This study had some limitations. 1. We didn’t investigate the change of learning and memory ability after infrasound exposure. However, our past study revealed that the 16 Hz, 90 dB infrasound-induced disorders of mitochondria and microtubules were gradually alleviated from the 7th d after infrasound exposure[71]. Therefore, we speculate that the impairment of learning and memory induced by infrasound may be temporary. 2. In present study, we only assessed the effect of infrasound on spatial learning and memory. The reason is that hippocampus, the center of spatial learning and memory, is especially vulnerable to infrasound [8, 25, 72]. Currently, there are no researches on the effect of infrasound on other kinds of learning/memory (such as recognition memory). We will investigate this point in the future. 3.Astrocytes in cultured medium have different external environment from astrocytes in vivo. Although infrasound possesses the characteristics of strong penetration and low-attenuation, the cultured astrocytes may exhibit different responses to infrasound exposure with astrocytes in vivo. Co-culture of astrocytes and neurons or acute hippocampal slices may be preferable.

Infrasound also has negative effect on the hearing and/or vestibular function. A case study revealed that, for 46 interviewed employees who were exposed to infrasound, 14 employees reported auditory symptoms and 9 employees reported vestibular symptoms [73]. Animal experiments also found that infrasound injuried the ultrastructures of vestibular end-organs and increased the threshold of audibility in guinea pigs [74, 75].

According to previous studies, a car produced 12–16 Hz, 110–130 dB infrasound when traveling at high speed [76]. Thus, the infrasound parameter was set to 16 Hz, 130 dB in present study. In the real world, the infrasound exposure may be a longer-lasting sound exposure. However, the exposure time in most studies was about 7–30 d. In the future, we will investigate the long-term effects on the animals (2 months or more).

In summary, we identified a mechanism by which infrasound impairs learning and memory ability, involving the sequential stimulation of cytokines (TNF-α, IL-1β and IL-6), leading to a further increase in astroglial Cx43 HC opening, resulting in the excessive release of glutamate and ATP. Alternative pharmacological strategies based on this process could be used to preserve learning and memory ability after infrasound exposure.

### Supplementary Information

**Additional file 1: Figure S1.** In normal rats, siRNA targeting Cx43 down-regulated about 30% Cx43 mRNA level in hippocampus

**Additional file 2: Figure S2.** GFAP staining showed that the purity of cultured astrocytes exceeded 95%.

## Data Availability

All data can be available on the inquiry for the corresponding authors.
