# Supplementary figures and images for: Inhibition of astroglial hemichannels ameliorates infrasonic noise induced short-term learning and memory impairment
Source: Behav Brain Funct. 2023 Dec 18;19:23. doi: 10.1186/s12993-023-00226-7 (PMC10726613; doi:10.1186/s12993-023-00226-7)

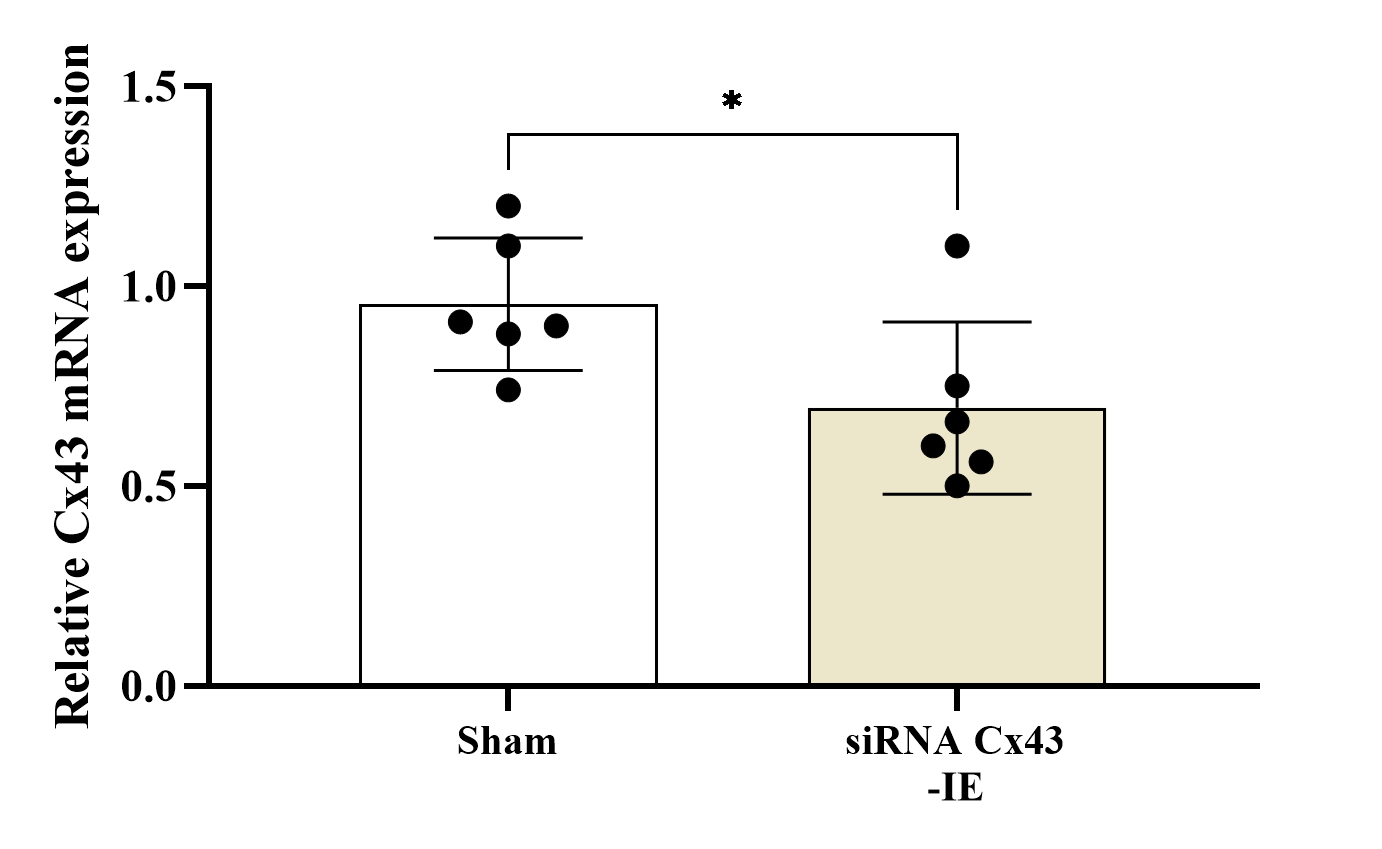

Supplement: Supplementary file 1 — Additional file 1: Figure S1. In normal rats, siRNA targeting Cx43 down-regulated about 30% Cx43 mRNA level in hippocampus [file 12993_2023_226_MOESM1_ESM.tif]

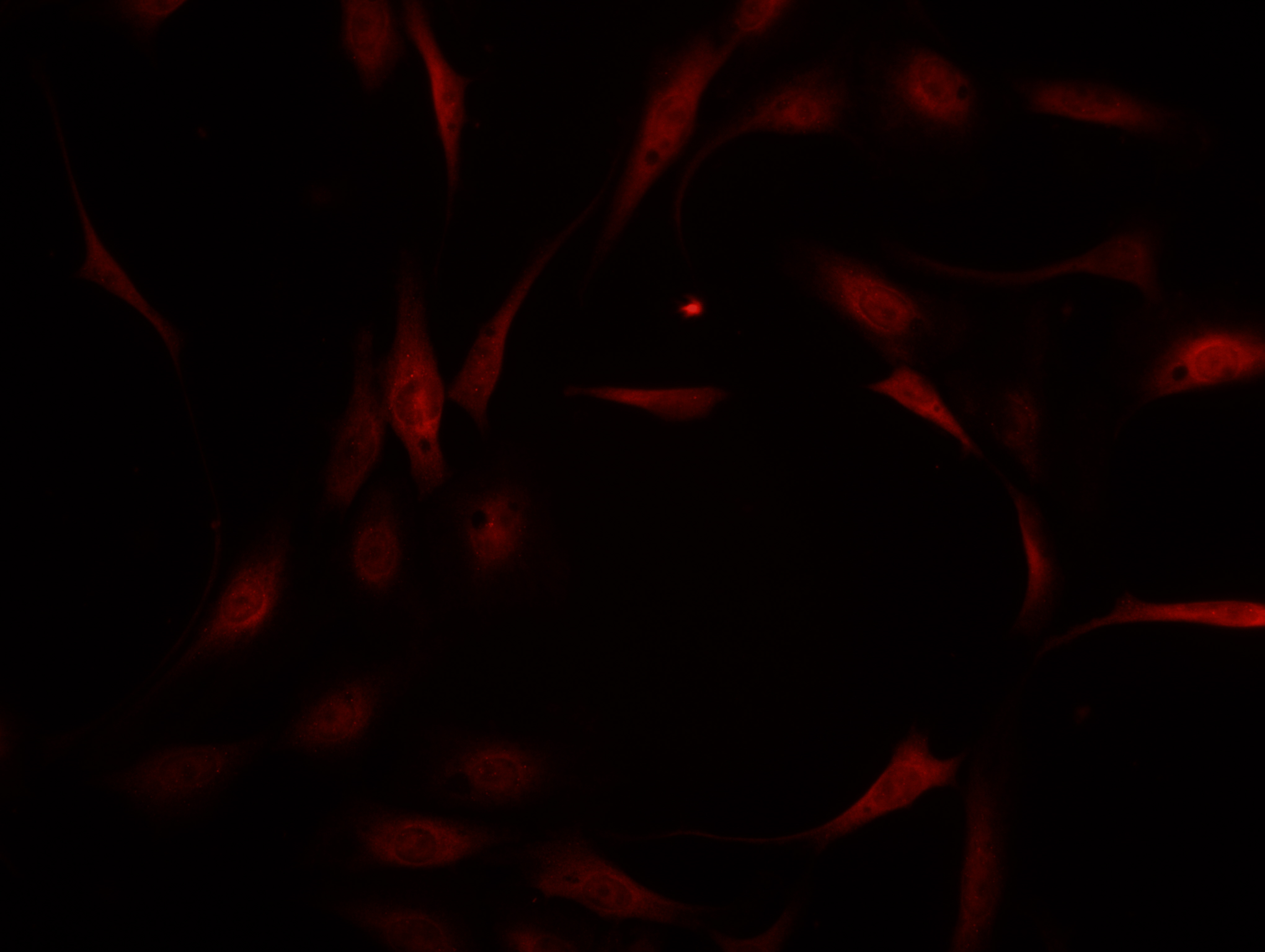

Supplement: Supplementary file 2 — Additional file 2: Figure S2. GFAP staining showed that the purity of cultured astrocytes exceeded 95%. [file 12993_2023_226_MOESM2_ESM.jpg]
